# Chickpea Can Be a Valuable Local Produced Protein Feed for Organically Reared, Native Bulls

**DOI:** 10.3390/ani11082353

**Published:** 2021-08-09

**Authors:** Francesco Serrapica, Felicia Masucci, Giuseppe De Rosa, Serena Calabrò, Claudia Lambiase, Antonio Di Francia

**Affiliations:** 1 Dipartimento di Agraria, Università di Napoli Federico II, Via Università 100, 80055 Portici, Italy; francesco.serrapica@unina.it (F.S.); giderosa@unina.it (G.D.R.); claudia.lambiase@unina.it (C.L.); antonio.difrancia@unina.it (A.D.F.); 2 Dipartimento di Medicina Veterinaria e Produzioni Animali, Università di Napoli Federico II, Via Delpino 1, 80137 Napoli, Italy; serena.calabro@unina.it

**Keywords:** native breeds, growing bulls, pulse seeds, metabolic profile, organic meat production

## Abstract

**Simple Summary:**

We aimed to demonstrate the benefits of using chickpea as energy-protein feed in diets for bulls belonging to a native Italian breed (Maremmana) organically reared. Compared to the control diets containing barley, the dietary inclusion of chickpea improved the growth rate and carcass conformation of bulls, reduced the feeding costs, and did not impair the plasma metabolic parameters and meat quality. These results suggest that chickpea may allow a sustainable performance improvement of native breeds within their traditional farming systems.

**Abstract:**

We assessed the effects of inclusion of chickpea from 24 to 21%, as feed basis, in diets for organically reared bulls. Sixteen young bulls (270 ± 6.4 days of age; 246 ± 0.13 kg in weight) belonging to a native Italian breed (Maremmana) were randomly assigned to two dietary treatments. The control diets were based on mixed grass hay, maize meal, and barley meal. In the experimental diets, barley was equally substituted by locally produced chickpea. Animals were weighed every 2 weeks until the prefixed slaughtering weight (630 kg). Plasma metabolites were measured at the 1st, 7th, and 14th month of the experiment. Chemical composition, colour, shear force, and water holding capacity of meat were assessed on *Longissimus thoracis et lumborum* 7 days after slaughter. The chickpea-fed animals showed a significantly greater average daily gain (1064 vs. 1168 kg/day), a shorter growing phase (364 vs. 335 days), and a better carcass conformation. Plasma metabolites and meat quality were not influenced by the treatments. The better growth performance and carcass quality of the chickpea fed bulls resulted in a higher economic profit for the chickpea-based diets. Results suggest that chickpea may allow sustainable performance improvement of native breeds within their traditional farming systems.

## 1. Introduction

**Figure 1:**
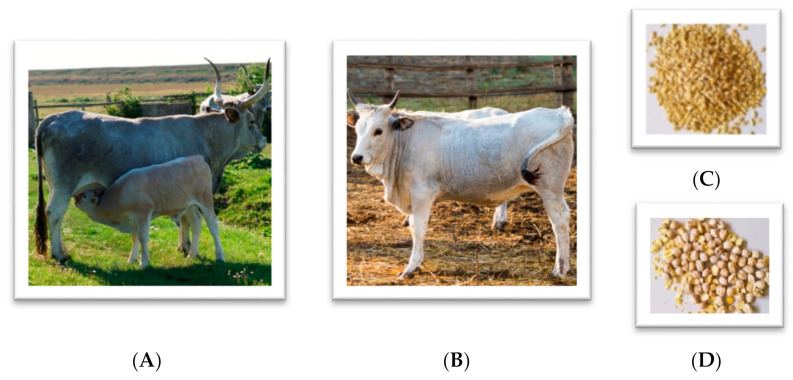
After weaning through an extensive cow-calf system (**A**), Maremmana young bulls (**B**) were fed growing and finishing diets containing feed-grade barley (**C**) or chickpea (**D**).

As a result of agriculture industrialization, several European native cattle breeds have suffered a dramatic decrease in numbers in favour of a few, highly selected, more productive breeds [1]. All the same, several local cattle continue to be farmed in specific areas of Europe where they support rural economies and, when extensively managed, enhance the agricultural landscape and provide generally safe and animal welfare-friendly foods [2,3,4]. This is the case of some Italian native cattle such Maremmana and Podolica that are extensively reared for meat production in semi-natural, hilly, and mountainous landscapes of central and southern Italy. These breeds are well adapted to constraints of these environments thanks to the massive skeletal structure, leg solidity, and hard hooves jointly to the remarkable parasite resistance, and the excellent maternal aptitude [5,6,7]. The calves free-range with their mother on the poorly nutritious shrubs of Mediterranean maquis until weaning, at about 8–10 months of age, when they are confined into feedlots until slaughter [8,9] (Figure 1A,B). As this production system is easily adaptable to organic rules, several farms rearing these cattle have converted to organic production [10,11]. Besides the use of local animal genetic resources, large use of pasture and forage, and the strong restrictions to medical drugs, organic livestock farming is characterized by reliance mainly on home-grown feed resources rather than on external input [12,13]. According to this rule, in many extensive beef organic farms the growing bulls are fed home-grown forage and cereals with scarce or no use of external, expensive organic protein sources [14,15]. Dietary protein deficiencies that may easily result from such a feeding regime might be overcome by producing pulse seeds that, besides providing feed protein, can also raise nitrogen and organic matter in the soil and alleviate the weed and pest problems related to the repeated cultivation of cereals [16,17,18].

Chickpea cropping (*Cicer arietinum* L, kabuki types) is attractive in Mediterranean context because of its low input requirement and resistance to drought joined to a long sowing window [19]. Although it is mainly produced for human consumption, feed grade chickpeas can be an energy-protein feedstuff alternative to cereals due to the higher metabolizable energy content and the protein content is almost double [20,21,22]. A restriction of the use of chickpea in ruminant feeding could be the large share of rumen degradable protein [23] that, if exceeding the amount required from rumen bacteria, might determine nitrogen loss from rumen and impair feeding efficiency and feed conversion ratio [24,25]. It has been hypothesised that chickpea can be a suitable energy-protein feed for young bulls. Hence, this trial aimed to evaluate the effects of dietary inclusion of chickpea on performance, carcass characteristics, and meat quality of Maremmana young bulls organically farmed. The costs and incomes associated to each dietary treatment were also evaluated.

## 2. Materials and Methods

The study was carried out in an organic farm rearing Maremmana cattle in Lazio, a region of Central Italy. Before and throughout the study the animals were managed in similar way in terms of feeding and management according to the legislation on organic livestock farming [12].

### 2.1. Experimental Design, Animals, and Diets

Sixteen Maremmana weaned young bulls (on average, 270 ± 6.4 days of age; Figure 1B) were weighed (246 ± 0.13 kg) and randomly allocated into the two groups barley and chickpea, homogenous for number, age, and body weight (BW). The slaughtering weight was set at 630 kg and the trial lasted 14 months during which three growing diets (until 300 kg; 300–400 kg; 400–500 kg) and one finishing (500–until 630 kg) diet were formulated according to the energy needs indicated by Institut national de la recherche agronomique (INRA, [26]).

According to the feeding plan usually adopted in the study area, the control diets were based on mixed grass hay, maize meal, and barley meal at 116 g/kg of crude protein (CP) on a dry matter (DM) basis. In the chickpea diets, barley was equally replaced for chickpea at 223 g/kg DM of CP (Table 1). All concentrates were fed grade quality (Figure 1C,D).

Barley (cultivar Explora) and chickpea (cultivar Principe) were locally produced (5.9 and 2.1 kg/ha respectively for barley and chickpea). The concentrates (i.e., maize and barley or chickpea) were ground and fed twice daily (h 0800–1500) at the established amount, while the hay was supplied ad libitum (on average, 5% orts). The animals were individually housed into adjacent pens with concrete solid floor and straw bedding equipped with feed manger and water bowls. 

### 2.2. Experimental Measures and Sampling Procedure 

Each animal was weighed at group formation and thereafter at 2-week intervals along with measurement of the hay intake calculated as the difference between hay offered and refused. At the 1st, 7th, and 14th months, coinciding with the farm official sanitary routine inspections, blood samples of each animal were collected into Li-heparinized vacuum tubes (Vacutainer, Becton Dickinson Italia S.p.a., Milano, Italy) by coccygeal venipuncture performed by a skilled and authorized veterinarian. The samples were immediately cooled in a thermo-isolate icebox and centrifuged (3000 rpm × 15 min at 4 °C) on the field within 30 min. Recovered plasma was transported to the laboratory on dry ice, and then stored at −20 °C until analysed. At the target weight (630 kg), the animals were deprived of feed but not of water for 12 h, transported to the abattoir, and then weighed, stunned, and slaughtered according to the EU Regulation. After dressing and 24 h chilling at 4 °C, the carcasses were weighed, visually scored according to the EUROP classification for conformation (E excellent; U very good; R good; O fair; P poor) and fatness (1 low; 2 slights; 3 medium; 4 high; 5 very high), and *Longissimus thoracis et lumborum* (LT) muscle was removed from the right side of each carcass. In the meat laboratory, the LT muscles were cut into 3–4 cm slices, vacuum packaged, matured at 4 °C until 7 days post-mortem, and then analysed. 

### 2.3. Chemical and Instrumental Analyses 

The feed samples were analysed according to the methods of Association of Official Analytical Chemists (AOAC, [27]) to determine DM (procedure 930.15), ether extract (EE, procedure 954.02), ash (procedure 942.05), and CP (Nitrogen × 6.25; procedure 976.05) contents. Neutral detergent fibre exclusive of residual ash (NDFom) and acid detergent fibre (ADF) were determined by the methods of Van Soest et al. [28], using alfa-amylase and sodium sulphite for NDF. Acid Detergent Lignin (ADL) was determined by digesting the ADF residue with 72% sulfuric acid solution [29]. Starch was determined according to the Ewers’ method [30] by using a Polax-2L polarimeter (Atago Co., Ltd., Tokyo, Japan) in 200-mm-long observation tubes. Soluble protein (SP) and non-protein nitrogen (NPN) were determined as described by Licitra et al. [31]. 

Plasma samples were assayed for glucose, total cholesterol, triglycerides, total protein, urea, creatinine, ß-hydroxybutyric acid (BHBA), alanine aminotransferase (ALT), aspartate aminotransferase (AST), and alkaline phosphatase (AP). Analyses were performed by using standard commercial kits and a UV spectrophotometer (Jasco V-530, Jasco, Tokyo, Japan) adopting the kits manufacturer recommended procedures (Sentinel Chemical, Milan, Italy). 

The meat samples were analysed (three replicates/sample) for chemical composition (protein, fat, ash) and total and insoluble collagen according to AOAC [27] and Modzelewska-Kapituła and Nogalski [32], respectively. Meat colour was assessed on cylindrical samples (1 cm × 1 cm cross section and 2 cm long) according to the CIE system using a spectrophotometer U-3000 (Hitachi, Tokyo, Japan) with D65 illuminant as previously described [33]. Gravimetric method was used to determine drip loss of raw meat preserved at 5 °C for 48 h and cooking loss of vacuum-packed samples cooked in water bath at 75 °C for 50′ according to the procedure described in Braghieri et al. [34]. Shear force on raw and cooked meat (samples of 1 cm × 1 cm cross section and 2 cm long) was determined using a Warner Bratzler Share apparatus on Instron 5565 as described by Marrone et al. [35].

### 2.4. Economic Analysis

An input–output budgeting procedure was used to calculate the costs and incomes associated to the two dietary treatments. The actual farm gate-price was used for both inputs and outputs. The feed costs (€/kg as fed) were mixed grass hay 0.12, maize meal 0.26, barley 0.22, chickpea 0.26, vitamin and mineral premix 1.50, and refer to feed-grade and organic certified feedstuffs. The amount of feedstuff consumed, the labour cost, and the other variable costs were calculated for each animal based on the length in days of each growing phases. Labour cost per head was computed based on an hourly wage of 7.30 € and 3 min of labour per day. Daily costs for machinery and health and hygiene products and services were estimated at 0.12 and 0.06 €/head, respectively. The interests on working capital were based on a 5% money legal interest rate. The cost per kg of weight gain was calculated by dividing total costs by total weight gain. Income achieved per animal was computed based on the selling value of carcass at slaughter, which varied according to carcass weight and conformation. The EU subsidies (60.26 €/head [36]) were also added. The economic margin was calculated as the differences between the income and the above-mentioned costs. Finally, to compare the economic efficiency of each treatment under different market scenarios, a break-even price analysis was performed to determine the maximum price of chickpea and barley at which a non-negative economic margin can be reached.

### 2.5. Calculation and Statistical Analysis

The net energy for maintenance and growth (ENmg) and the intestinal digestible protein fractions (PDIA, PDIE and PDIN) values of the diets were estimated based on the chemical composition of the feedstuffs [26]. Data were analysed with SAS software (SAS Institute, 1991, Cary, NC, USA). Before analyses, the Shapiro–Wilk’s and Levene’s tests were used to test the normality of distribution and homogeneity of variance, respectively. The average daily gain (ADG) at each phase was calculated as the difference between initial and final BW divided by the number of growing days. The bull was used as the experimental unit for all analyses. Plasma analyses underwent analysis of variance for repeated measures with diet as a non-repeated factor and week of observation and week of observation × treatment as repeated factors. Data of ADG, slaughtering traits, and meat quality were analysed by one-way analysis of variance with diet as factor. Carcass conformation grades were numerically transformed (5 is the fleshiest and 1 is the thinnest) and, as for fatness scores, analysed by the Kruskal–Wallis test with diet as a factor. 

## 3. Results

The barley and chickpea diets had a roughly similar energy content while the CP levels were constantly higher for the chickpea diets (Table 2). The PDIN and PDIE levels were well balanced in the chickpea diets (i.e., PDIN to PDIE ratios 1.0), whereas PDIN were constantly lower than PDIE in the barley diets (PDIN to PDIE ratio 0.85). Compared to the needs [26], both dietary treatments slightly exceeded the energy requirements, whereas the supplies of intestinal digestible protein were slightly higher in the sole chickpea-based diets (supply to needs ratio on average 1.1). 

The effects of the dietary inclusion of chickpea on the animal growth performances are in Table 3. The ADG of the two dietary groups did not differ at the beginning (phase 1, until 300 kg) and at the end (finishing phase, BW 500 kg-slaughtering) of the trial, whereas the chickpea group showed significantly better ADG values at phases 2 (BW 300–400 kg, *p* < 0.05) and 3 (BW 400–500, *p* < 0.05), and, as an overall effect, for the total ADG (*p* < 0.001). Consistently, the length of phase 1 and of the finishing phase did not differ among the groups, while phase 2 (*p* < 0.05), phase 3 (*p* < 0.05), and the total length of the growing period (from 300 to 630 kg; *p* < 0.01) were shorter for the chickpea-fed animals that also showed younger age at slaughtering (*p* < 0.001). 

The concentrations of plasma metabolites are summarized in Table 4. No significant interaction treatment × week of observation was found. The concentration of some metabolites defining the energy (i.e., glucose, total cholesterol, and BHBA) and protein (i.e., total protein, urea, and creatinine) status, and the levels of circulating enzymes (ALT, AST, and AP) and electrolytes were influenced by the time of sampling (*p* < 0.05) according to the age-related trend observed for growing bulls [37,38,39].

The plasma concentration of urea and creatinine and AP activity were higher (*p* < 0.05) in chickpea-fed bulls, whereas total protein was unaffected by the diet. The total cholesterol was significantly lower in the chickpea group (*p* < 0.05), while no differences were observed for glucose and triglycerides as well as for the blood activity of circulating liver enzymes, and electrolytes concentration. 

The slaughtering traits and meat quality as influenced by the diet are shown in Table 5. Carcass weight and dressing percentage (carcass weight/live weight × 100) did not differ between the two dietary groups, whereas the carcasses from the chickpea-fed bulls were significantly better conformed than those from the barley group (*p* < 0.01) but had greater (*p* < 0.01) fatness score. Meat quality was unaffected by the diet, except drip loss was higher in the barley group (*p* < 0.01), and cooking loss was higher in the chickpea group (*p* < 0.01). 

The economic performances associated to each dietary treatment are given in Table 6. The feeding costs were lower (−4.4%) for the chickpea-fed bulls due to the shorter growing period and, thus, to the lower feed consumption. Likewise, also the labour, machinery and health/hygiene costs were smaller (−8.4%), since they were related to the length of the growing period. As a result, the weight gain cost was lower (−6.2%) for the chickpea-based diets. The income per carcass was 11.7% greater for the chickpea-fed bulls, due to the better conformation score. This extra income had a larger positive impact than the reduction of the feeding costs, whose magnitude tied to the shorter growing period was partially offset by the higher chickpea price (0.22 vs. 0.26 €/kg respectively for barley and chickpea). As an overall effect, the use of chickpea resulted in a higher economic margin net of the EU subsidies (+183.5 €/carcass). It should be noted that the EU subsidies constitute the largest share of the profit for the barley treatment (94% of the economic margin versus 24% for the chickpea-based diets). The break-even analysis set the price to reach costs recovery point at 0.88 €/kg (+238.5% of actual price) for chickpea and 0.31 (+40.9% of actual cost) for barley.

## 4. Discussion

Organic and locally produced chickpea presents two main benefits compared to similarly produced barley: It is richer in protein, so it can help meet the animals’ protein requirement according to the organic standards, and fixes atmospheric nitrogen, so contributing to reduced N_2_O emissions and mitigating the carbon footprint of cropping systems [40]. In this study, we aimed to demonstrate the benefits of using chickpea as energy-protein feed in diets for native bulls in terms of growth performance and economic return. The substitution of barley for chickpea in practical-type diets resulted in both a higher growth rate and a shorter growing period most likely driven by the higher and well-balanced dietary protein level [41]. By contrast, the worse performance of barley-fed bulls may be ascribed to the lower nitrogen supply for microbial syntheses in the rumen (PDIN to PDIE ratio 0.85) that resulted in a lower protein availability at the intestinal level [42,43].

As reviewed by Bampidis and Christodoulou [22], the scarce studies on the use of chickpeas on growing ruminants were performed by comparing chickpeas with other pulse seeds, mostly soybean, in isonitrogenous and isoenergetic diets. Thus, it is not surprising that, in contrast with our results, little or no effects related to the dietary use of chickpeas were detected. However, these reports may indirectly support our assumption that the higher protein of chickpea diets combined with the unbalanced protein supply of the barley diets may be the drivers of better growth performance of the chickpea group. The better ADG may also indirectly indicate that anti-nutritional compounds present in chickpeas (e.g., protease inhibitors and lectins) were inactivated by rumen fermentations. Cutrignelli et al. [44] observed a reduced growth rate after weaning in 4-month-old Marchigiana calves fed faba bean. Indeed, in the present study, the older age of the animals (about 9 months) has meant a fully developed rumen able both to inactivate anti-nutritional factors and to efficiently utilize the nitrogen from degraded dietary protein [45]. Haematological variables can provide useful indications about physiological changes and nutritional status of animals [46], and, albeit some differences were observed between groups, the values were always within the range for native bulls [47,48], further confirming the good health status of animals. Few data are available on the effects of pulse grains on blood profile of beef cattle and none of them addressed the use of chickpea [49]. In our previous study, the isoenergetic and isonitrogenous replacement of soybean cake by chickpea in diets for primiparous dairy buffaloes did not influence cholesterol concentration, whereas in the present report the chickpea-fed bulls showed a lower plasma cholesterol level [50]. A large body of evidence [47,51,52,53] indicates an inverse relationship between protein intake and blood cholesterol concentration, suggesting that the quantity and possibly the source of dietary protein influence lipid metabolism in ruminants [54]. The increased plasma urea concentration in the chickpea-fed group was expected since it reflects the higher protein intake, and possibly more soluble and rumen degradable chickpea protein [23]. Nevertheless, chickpea can be considered a good source of metabolizable energy and protein, as denoted by the higher growth rate of chickpea-fed bulls and, accordingly, by both higher plasma creatinine and AP levels. Creatinine is a product of the skeletal muscles’ metabolism. Blood creatinine concentration is strictly related to the growth rate of the muscular tissue and, thus, may vary according to animal live weight gain [55]. Similarly, the higher activity of AP indicates a higher growth potential in chickpea-fed animals, since this enzyme is related to bone growth [56].

The ultimate measurement of the efficiency of a diet for beef cattle is not only given by weight gain, but also by the effects on carcass quality. Theoretically, a higher dietary protein level might result in greater muscle development [41] and indeed chickpea-fed bulls showed better carcass conformation, but also a higher fatness score. A major adiposity in carcasses has been also observed in chickpea-fed lambs [57] and it could be due to a decrease of acetate to propionate ratio as reported in chickpea fed steers [58,59]. The major molar proportion of propionate could likely have promoted the synthesis of fatty acids of the adipose tissue [60]. 

The values observed for meat quality parameters were in line with previous reports for Maremmana bulls of comparable age [61,62,63,64] and were not substantially influenced by the diet. In particular, no differences were observed for tenderness although a higher growth rate might result in meat tenderness benefits tied to a faster protein turnover and a lower amount of aged protein and firm muscle links [65,66]. Similarly, the higher carcass fatness did not influence meat lightness [67] since chickpea diet only influenced covering fat but not intramuscular fat, as it is shown by the lack of significant differences for the meat fat content. 

The dietary inclusion of chickpea increased the income per head due both to the reduction of feeding costs and to the higher price paid for the better-conformed carcass, namely the main drivers of beef farm profitability [68]. The European subsidies allocated through the Community Agricultural Policy had a lower impact on the profitability of the chickpea-based system. This is a further strength of this feeding system since these subsidies are expected to be reduced in the long term, thus further increasing the economic instability of the traditional cereal-based feeding system [69]. Despite these strengths, the scenario analysis for the use of chickpea must be well thought-out. Currently, European chickpea production is still low and goes for human consumption [70]. Unlike other legumes such as peas, whose food market quickly saturated, allowing a surplus available for livestock feeding, food demand for chickpeas is expected to increase, resulting in potential price increments [70]. Nevertheless, even under increased chickpea price volatility, the barley-based diets still appear the weakest feeding strategy. Indeed, as sensitivity analysis highlighted, the break-even point for the non-negative income was reached at 238.5% increment of the chickpea price, indicating the robustness of chickpea treatment up to extreme price increases. 

## 5. Conclusions

Substitution of barley for chickpea in diets for young native bulls improved the growth rate and carcass conformation, reduced the feeding costs, and did not impair the plasma metabolic parameters and meat quality. Overall, the introduction of chickpea in amount ranging from 24 to 21%, as feed basis, appears to guarantee better long-term economic sustainability of meat organic production by using native cattle. We conclude that chickpea represents an attractive energy-protein feed, allowing sustainable performance improvement of native breeds within their traditional farming systems and so contributing to the proper management and conservation of semi-natural habitats as well as to agroecological production of animal foods. 

## Figures and Tables

**Table 1:** Chemical composition and nutritional characteristics (g/kg of dry matter, DM, if not otherwise stated) of the feeds included in the diets.

Item	Mixed Grass Hay	Maize Meal	Barley Meal	Chickpea Meal
Chemical composition				
Dry matter, g/kg as-feed	850.0	864.0	867.0	890.0
Ash	84.7	14.0	26.0	26.0
Crude protein	127.0	94.0	116.0	223.0
SP	44.5	16.1	20.5	154.8
NPN	30.5	10.7	7.5	60.9
Ether extract	23.5	43.0	21.0	51.7
NDFom	591.0	120.0	216.0	257.0
ADF	321.0	30.0	63.0	100.0
ADL	70.9	6.0	11.0	28.0
Starch	-	64.1	52.2	44.9
Nutritional characteristics ^1^				
PDIA	40.0	53.2	39.2	19.5
PDIE	91.8	97.2	116.5	90.0
PDIN	84.7	74.	91.1	132.0
NEmg, MJ/kg DM	5.5	9.4	8.8	9.4

^1^ Values computed according to INRA tables [26]. SP, soluble protein; NPN, non-protein nitrogen; NDFom, Neutral Detergent Fibre excluding residual ash; ADF, Acid Detergent Fibre; ADL, acid detergent lignin; PDIA, protein undegraded in the rumen but truly digestible in the small intestine; PDIE, true intestinal digestible protein, when fermentable energy is the limiting factor; PDIN, true intestinal digestible protein, when fermentable N is the limiting factor; NEmg, net energy for maintenance and growth.

**Table 2:** Ingredients, chemical composition, and nutritional characteristics of the diets through the growing and finishing phases.

Item	Phase 1 ^1^		Phase 2 ^1^		Phase 3 ^1^		Phase 4 ^1^	
	Barley	Chickpea	Barley	Chickpea	Barley	Chickpea	Barley	Chickpea
**Requirements**								
ENmg, MJ/day	34.3	34.3	41.9	41.9	48.8	48.8	56.4	56.4
PDI, g/day	473	473	551	551	631	631	722	722
**Ingredients**, kg/day								
Mixed grass hay	3.8	3.8	4.5	4.5	5.0	5.0	5.5	5.5
Maize meal	1.2	1.2	1.5	1.5	2.0	2.0	2.3	2.3
Barley meal	1.6	-	1.8	-	2.0	-	2.2	-
Chickpea meal	-	1.6	-	1.6	-	2.0	-	2.2
Vitamin-Mineral Mix ^2^	0.30	0.30	0.40	0.40	0.45	0.45	0.50	0.50
**Chemical composition**, g/kg DM								
Ash	98.9	98.4	103.9	103.4	101.2	104.3	100.8	100.4
Crude protein	114.4	137.9	111.5	135.6	110.8	134.1	110.6	133.6
SP	31.7	63.6	31.5	61.7	30.9	60.0	30.8	59.6
NPN	20.9	32.8	20.1	32.1	19.8	31.3	19.7	31.1
Ether extract	25.2	32.5	25.2	32.12	25.8	32.5	26.0	32.6
NDFom	392.2	400.9	389.2	397.4	379.4	354.2	376.8	384.7
ADF	199.4	202.2	193.0	200.6	187.1	181.9	185.5	192.8
ADL	42.1	46.0	41.9	45.5	40.6	38.5	40.2	43.7
Starch	269.8	248.5	268.7	248.6	285.3	265.8	289.5	270.2
**Nutritional characteristics ^3^**								
PDIA, g/d	238.8	217.2	283.2	253.2	330.0	346.0	367.6	331.0
PDIE, g/d	558.8	525.4	658.8	621.2	760.0	746.0	844.0	798.4
PDIN, g/d	476.8	538.4	562.2	631.5	646.0	748.0	717.0	801.7
NEmg, MJ/d	39.6	40.8	48.1	46.8	56.2	54.7	62.6	60.9

^1^ Phase 1: until 300 kg BW; Phase 2: 300–400 kg BW; Phase 3: 400–500 kg BW; Phase 4; 500–630 kg BW. ^2^ Containing (per kg, based on the manufacturer’ declared content): 4,000,000 IU of vitamin A; 100,000 IU of vitaminD3; 1500 mg of vitamin E; 1400 mg of vitamin B6; 1400 mg of vitamin C; 1100 mg of vitamin B1; 500 mg of vitaminB2; 5000 mg of choline chloride; 1000 mg of biotin; 800 mg of pantothenic acid; 700 mg of niacinamide; 180 g calcium;38 g of phosphorous; 70 g of sodium; 15 g of magnesium; 1000 mg of S as copper-II-sulfate; 1600 mg of Mn as manganese-II-oxide; 5400 mg of Zn as zinc sulfate, monohydrate; 80 mg of I as calcium iodate, anhydrous; and 10 mg of Se as sodium selinte. ^3^ Nutritional characteristics were calculated from the INRA tables [26]. NEmg, net energy for maintenance and growth; PDI, intestinal digestible protein; SP, soluble protein; NPN, non-protein nitrogen; NDFom, Neutral Detergent Fiber excluding residual ash; ADF, Acid Detergent Fiber; ADL, acid detergent lignin; PDIA, protein undegraded in the rumen but truly digestible in the small intestine; PDIE, true intestinal digestible protein, when fermentable energy is the limiting factor; and PDIN, true intestinal digestible protein, when fermentable N is the limiting factor.

**Table 3:** Growth performances (LSM ± SEM) of the bulls fed barley (control) or chickpea diets.

	Barley	Chickpea	SEM	*p*-Value
Feeding period length, day	364.3	335.6	4.3	0.0003
Until 300 kg	51.0	58.1	7.2	ns
300–400 kg	108.4	88.6	5.4	0.02
400–500 kg	92.9	75.5	4.6	0.018
500 kg-slaughtering	112.0	113.4	4.3	ns
Average daily gain, g/day	1064.0	1167.5	15.0	0.002
Until 300 kg	1112.5	975.0	99.2	ns
300–400 kg	962.5	1175.0	52.9	0.013
400–500 kg	1062.5	1325.0	72.8	0.02
500 kg–slaughtering	1300.0	1225.0	68.8	ns
Slaughtering age, day	635.1	606.1	4.9	0.0003

ns, not significant; LSM, least square mean; SEM, standard error of the means.

**Table 4:** Blood plasma metabolites (LSM ± SEM) as influenced by the diets and blood sampling time.

	Diet		Month of Experimental Period			Effect *p*		
	Barley	Chickpea	0	7	14	Diet	Month	D × M
Glucose, mmol/L	6.65 ± 0.10	6.54 ± 0.10	7.21 ± 0.07 ^a^	6.43 ± 0.07 ^b^	6.14 ± 0.07 ^b^	ns	0.039	ns
Total cholesterol, mmol/L	1.36 ± 0.05	1.25 ± 0.05	1.65 ± 0.05 ^a^	1.28 ± 0.05 ^b^	1.47 ± 0.05 ^b^	0.048	0.047	ns
Triglycerides, mmol/L	0.23 ± 0.02	0.20 ± 0.02	0.24 ± 0.01 ^a^	0.24 ± 0.01 ^a^	0.18 ± 0.01 ^b^	ns	ns	ns
Total protein, g/L	68.74 ± 2.36	66.35 ± 2.36	78.92 ± 1.22 ^a^	61.98 ± 1.22 ^b^	61.74 ± 1.22 ^b^	ns	0.034	ns
Urea, mmol/L	5.92 ± 0.10	6.13 ± 0.10	5.39 ± 0.12 ^a^	6.30 ± 0.12 ^b^	6.38 ± 0.12 ^b^	0.028	0.016	ns
BHBA, mmol/L	0.26 ± 0.10	0.28 ± 0.10	0.23 ± 0.01 ^a^	0.31 ± 0.01 ^b^	0.29 ± 0.01 ^b^	ns	0.026	ns
Creatinine, mmol/L	119.37 ± 2.05	124.74 ± 2.05	112.78 ± 3.02 ^a^	134.67 ± 3.02 ^b^	118.73 ± 3.02 ^a^	0.021	0.031	ns
Phosphorus, mmol/L	2.34 ± 0.13	2.25 ± 0.13	2.03 ± 0.06^a^	2.42 ± 0.06 ^b^	2.43 ± 0.06 ^b^	ns	0.032	ns
Calcium, mmol/L	2.10 ± 0.07	2.32 ± 0.07	1.58 ± 0.21 ^a^	2.01 ± 0.21 ^b^	3.05 ± 0.21 ^b^	ns	0.034	ns
AST, U/L	71.40 ± 2.17	72.69 ± 2.17	83.19 ± 0.71 ^a^	69.62 ± 0.71 ^b^	66.33 ± 0.71 ^c^	ns	0.0006	ns
ALT, U/L	23.94 ± 1.25	22.82 ± 1.25	20.32 ± 0.79 ^a^	23.43 ± 0.79 ^a,b^	26.41 ± 0.79 ^b^	ns	0.027	ns
AP, U/L	275.61 ± 2.51	303.32 ± 2.51	244.02 ± 14.01 ^a^	325.78 ± 14.01 ^b^	298.60 ± 14.01 ^a^	0.043	0.046	ns

BHBA, ß-hydroxybutyric acid; AST, aspartate aminotransferase; ALT, alanine aminotransferase; AP, alkaline phosphatase; ns, not significant; LSM, least square mean; SEM, standard error of the means. ^a^^,b,c^ Values within a row with different superscripts differ significantly at *p* < 0.05.

**Table 5:** Slaughtering and carcass traits and meat quality (LSM ± SEM) of the bulls fed barley (control) or chickpea diets.

	Barley	Chickpea	SEM	*p*-Value
Carcass traits				
Carcass weight, kg	329.5	332.0	6.8	ns
Dressing percentage, %	52.0	52.1	1.1	ns
Carcass conformation ^1^	2.4	3.0	-	0.009
Carcass fat score ^2^	2.5	3.5	-	0.006
Ultimate pH	5.8	5.9	0.1	ns
Meat composition, %				
Dry matter	24.7	24.3	0.2	ns
Fat	2.7	2.3	0.2	ns
Ash	1.1	1.1	0.1	ns
Crude protein	21.0	20.9	0.2	ns
Total collagen	3.8	4.1	0.4	ns
Insoluble collagen	2.9	2.6	0.1	ns
Meat Color				
L* Lightness	40.0	39.4	1.1	ns
a*, red—green	18.9	18.1	0.3	ns
b*, yellow—blue	12.8	11.7	0.7	ns
Water holding capacity, %				
Drip loss	1.6	0.8	0.1	0.003
Cooking loss	24.3	29.1	0.7	0.002
Warner Bratzler Shear Force, kg				
Raw meat	3.5	3.1	0.2	ns
Cooked meat	6.7	6.3	0.5	ns

^1^ 1 = poor to 5 = excellent. ^2^ 1= minimum to 5 = maximum; ns, not significant; LSM, least square mean; SEM, standard error of the means.

**Table 6:** Economic performance (€/head if not otherwise stated) of the dietary treatments including barley (control) or chickpea.

	Barley	Chickpea	Δ ^1^
A. Feeding costs	756.6	724.5	32.1
Mixed grass hay	210.7	194.1	16.6
Maize meal	172.8	159.4	13.4
Barley/Chickpea	155.4	169.4	−14.0
Vitamin-Mineral Mix	217.7	201.6	16.1
B. Other costs	200.6	184.9	15.7
Health and Hygiene	22.7	20.9	1.8
Machinery	45.4	41.8	3.6
Labor costs	132.5	122.2	10.3
C. Interest of working capital ^2^	47.9	45.5	2.4
D. Total costs (A + B + C)	1005.1	954.9	50.2
E. Cost of weight gain ^3^, €/kg	2.6	2.4	0.2
F. Total income	1069.3	1202.6	−133.3
Carcass selling price	1009.0	1142.3	−133.3
Subsidies	60.3	60.3	0.0
G. Economic margin (F-D)	64.2	247.7	−183.5
H. Break-even price ^4^, €/kg	0.31	0.88	−0.57

^1^ Calculated as difference between the control and chickpea treatments. ^2^ Calculated by multiplying the legal monetary rate (5%) by variable costs (i.e., feeding costs + other costs). ^3^ Calculated by dividing total costs by total weight gain. ^4^ Projected market price at which the total cost equals the economic margin.

## Data Availability

The datasets of the present study are available from the corresponding author on reasonable request.

## References

[1] Food and Agriculture Organization (FAO) (2015). The Second Report on the State of the Word’s Animal Genetic Resources for Food and Agriculture.

[2] Zanoli R., Scarpa R., Napolitano F., Piasentier E., Naspetti S., Bruschi V. (2013). Organic Label as an Identifier of Environmentally Related Quality: A Consumer Choice Experiment on Beef in Italy. Renew. Agric. Food Syst..

[3] Bragaglio A., Napolitano F., Pacelli C., Pirlo G., Sabia E., Serrapica F., Serrapica M., Braghieri A. (2018). Environmental Impacts of Italian Beef Production: A Comparison between Different Systems. J. Clean. Prod..

[4] Bragaglio A., Braghieri A., Pacelli C., Napolitano F. (2020). Environmental Impacts of Beef as Corrected for the Provision of Ecosystem Services. Sustainability.

[5] Moioli B., Steri R., Marchitelli C., Catillo G., Buttazzoni L. (2017). Genetic Parameters and Genome-Wide Associations of Twinning Rate in a Local Breed, the Maremmana Cattle. Animal.

[6] Cosentino C., D’Adamo C., Naturali S., Pecora G., Paolino R., Musto M., Adduci F., Freschi P. (2018). Podolian Cattle: Reproductive Activity, Milk and Future Prospects. Ital. J. Agron..

[7] Foggi G., Ciucci F., Conte M., Casarosa L., Serra A., Giannessi E., Lenzi C., Salvioli S., Conte G., Mele M. (2021). Histochemical Characterisation and Gene Expression Analysis of Skeletal Muscles from Maremmana and Aubrac Steers Reared on Grazing and Feedlot Systems. Animals.

[8] Braghieri A., Pacelli C., Piazzolla N., Girolami A., Napolitano F. (2013). Eating Quality of Beef from Free-Range and Confined Podolian Young Bulls1,2. J. Anim. Sci..

[9] Fratini R., Riccioli F., Marone E. (2014). Cattle Breeding and Territory: A Survey on the Maremmana Breed Raised in Tuscany. Online J. Anim. Feed Res..

[10] Pauselli M. (2009). Organic Livestock Production Systems as a Model of Sustainability Development. Ital. J. Anim. Sci..

[11] Marino R., Albenzio M., Braghieri A., Muscio A., Sevi A. (2006). Organic Farming: Effects of Forage to Concentrate Ratio and Ageing Time on Meat Quality of Podolian Young Bulls. Livest. Sci..

[12] The Council of European Union (EC) (2010). Council Regulation (EC) No. 834/2007 of 28 June 2007 on organic production and labelling of organic products and repealing Regulation (EEC) No. 2092/91. Off. J. Eur. Union.

[13] Napolitano F., Braghieri A., Piasentier E., Favotto S., Naspetti S., Zanoli R. (2010). Effect of Information about Organic Production on Beef Liking and Consumer Willingness to Pay. Food Qual. Prefer..

[14] Sargentini C., Bozzi R., Lorenzini G., Degl’Innocenti P., Martini A., Giorgetti A. (2010). Productive Performances of Maremmana Young Bulls Reared Following Organic Rules and Slaughtered at 18 and 24 Months of Age. Ital. J. Anim. Sci..

[15] Braghieri A., Pacelli C., Bragaglio A., Sabia E., Napolitano F., Vastola A. (2015). The Hidden Costs of Livestock Environmental Sustainability: The Case of Podolian Cattle. The Sustainability of Agro-Food and Natural Resource Systems in the Mediterranean Basin.

[16] Monti M., Pellicanò A., Pristeri A., Badagliacca G., Preiti G., Gelsomino A. (2019). Cereal/Grain Legume Intercropping in Rotation with Durum Wheat in Crop/Livestock Production Systems for Mediterranean Farming System. Field Crops Res..

[17] Serrapica F., Masucci F., Romano R., Santini A., Manzo N., Seidavi A., Omri B., Salem A.Z.M., Di Francia A. (2020). Peas May Be a Candidate Crop for Integrating Silvoarable Systems and Dairy Buffalo Farming in Southern Italy. Agrofor. Syst..

[18] Calabrò S., Cutrignelli M.I., Gonzalez O.J., Chiofalo B., Grossi M., Tudisco R., Panetta C., Infascelli F. (2014). Meat Quality of Buffalo Young Bulls Fed Faba Bean as Protein Source. Meat Sci..

[19] Sellami M.H., Lavini A., Pulvento C. (2021). Phenotypic and Quality Traits of Chickpea Genotypes under Rainfed Conditions in South Italy. Agronomy.

[20] Mustafa A.F., Thacker P.A., McKinnon J.J., Christensen D.A., Racz V.J. (2000). Nutritional Value of Feed Grade Chickpeas for Ruminants and Pigs. J. Sci. Food Agric..

[21] Bampidis V.A., Christodoulou V., Nistor E., Skapetas B., Nistor G. (2009). The Use of Chickpeas (*Cicer arietinum*) in poultry diets: A review. Sci. Pap. Anim. Sci. Biotechnol..

[22] Bampidis V.A., Christodoulou V. (2011). Chickpeas (*Cicer arietinum* L.) in Animal Nutrition: A Review. Anim. Feed Sci. Technol..

[23] Sun B., Khan N.A., Yu P. (2018). Molecular Spectroscopic Features of Protein in Newly Developed Chickpea: Relationship with Protein Chemical Profile and Metabolism in the Rumen and Intestine of Dairy Cows. Spectrochim. Acta. A Mol. Biomol. Spectrosc..

[24] Illg D.J., Sommerfeldt J.L., Boe A.A. (1987). Chickpeas as a Substitute for Corn and Soybean Meal in Growing Heifer Diets1. J. Dairy Sci..

[25] Hadjipanayiotou M. (2002). Replacement of Soybean Meal and Barley Grain by Chickpeas in Lamb and Kid Fattening Diets. Anim. Feed Sci. Technol..

[26] Noziere P., Sauvant D., Delaby L. (2018). INRA Feeding System for Ruminants.

[27] AOAC (Association of Official Analytical Chemists) (2002). Official Methods of Analysis.

[28] Van Soest P.J., Robertson J.B., Lewis B.A. (1991). Methods for Dietary Fiber, Neutral Detergent Fiber, and Nonstarch Polysaccharides in Relation to Animal Nutrition. J. Dairy Sci..

[29] Robertson J.B., Van Soest P.J., James W.P.T., Theander O. (1981). The detergent system of analysis. The Analysis of Dietary Fibre in Food.

[30] International Organization for Standardization (ISO) (2000). Animal Feeding Stuffs. Determination of Starch Content–Polarimetric Method.

[31] Licitra G., Hernandez T.M., Van Soest P.J. (1996). Standardization of Procedures for Nitrogen Fractionation of Ruminant Feeds. Anim. Feed Sci. Technol..

[32] Modzelewska-Kapituła M., Nogalski Z. (2014). Effect of Gender on Collagen Profile and Tenderness of Infraspinatus and Semimembranosus Muscles of Polish Holstein-Friesian x Limousine Crossbred Cattle. Livest. Sci..

[33] Masucci F., De Rosa G., Barone C.M.A., Napolitano F., Grasso F., Uzun P., Di Francia A. (2016). Effect of Group Size and Maize Silage Dietary Levels on Behaviour, Health, Carcass and Meat Quality of Mediterranean Buffaloes. Animal.

[34] Braghieri A., Girolami A., Cifuni G.F., Riviezzi A.M., Pacelli C., Napolitano F. (2007). Shelf Life of Meat from Podolian Young Bulls in Relation to the Aging Method. J. Food Qual..

[35] Marrone R., Salzano A., Di Francia A., Vollano L., Di Matteo R., Balestrieri A., Anastasio A., Barone C.M.A. (2020). Effects of Feeding and Maturation System on Qualitative Characteristics of Buffalo Meat (*Bubalus bubalis*). Animals.

[36] European Parliament and Council of the European Union (2013). Regulation (EU) No 1307/2013 of the European Parliament and of the Council of 17 December 2013 Establishing Rules for Direct Payments to Farmers under Support Schemes within the Framework of the Common Agricultural Policy and Repealing Council Regulation (EC) No 637/2008 and Council Regulation (EC) No 73/2009. https://eur-lex.europa.eu/legalcontent/EN/TXT/?uri=CELEX%3A32013R1307&qid=1625337645078.

[37] Doornenbal H., Tong A.K., Murray N.L. (1988). Reference values of blood parameters in beef cattle of different ages and stages of lactation. Can. J. Vet. Res..

[38] Pavlík A., Filipčík R., Jelínek P., Bjelka M., Havlíček Z., Šubrt J., Šubrt J. (2008). Indicators of the Internal Environment of Beef Cattle during Fattening and their Correlation to the Quality of Beef. Acta Vet. Brno.

[39] Ndlovu T., Chimonyo M., Okoh A.I., Muchenje V., Dzama K., Raats J.G. (2007). Assessing the nutritional status of beef cattle: Current practices and future prospects. Afr. J. Biotechnol..

[40] Plaza-Bonilla D., Nogué-Serra I., Raffaillac D., Cantero-Martínez C., Justes É. (2018). Carbon Footprint of Cropping Systems with Grain Legumes and Cover Crops: A Case-Study in SW France. Agric. Syst..

[41] Prado I.N., Campo M.M., Muela E., Valero M.V., Catalan O., Olleta J.L., Sañudo C. (2015). Effects of Castration Age, Protein Level and Lysine/Methionine Ratio in the Diet on Colour, Lipid Oxidation and Meat Acceptability of Intensively Reared Friesian Steers. Animal.

[42] Hall M.B., Huntington G.B. (2008). Nutrient Synchrony: Sound in Theory, Elusive in Practice. J. Anim. Sci..

[43] Spanghero M., Mason F., Zanfi C., Nikulina A. (2017). Effect of Diets Differing in Protein Concentration (Low vs Medium) and Nitrogen Source (Urea vs Soybean Meal) on in Vitro Rumen Fermentation and on Performance of Finishing Italian Simmental Bulls. Livest. Sci..

[44] Cutrignelli M.I., Piccolo G., Bovera F., Calabrò S., D’Urso S., Tudisco R., Infascelli F. (2008). Effects of Two Protein Sources and Energy Level of Diet on the Performance of Young Marchigiana Bulls. 1. Infra Vitam Performance and Carcass Quality. Ital. J. Anim. Sci..

[45] Paduano D.C., Dixon R.M., Domingo J.A., Holmes J.H.G. (1995). Lupin (*Lupinus angustifolius*), Cowpea (*Vigna unguiculata*) and Navy Bean (*Phaseolus vulgaris*) Seeds as Supplements for Sheep Fed Low Quality Roughage. Anim. Feed Sci. Technol..

[46] Puppel K., Kuczyńska B. (2016). Metabolic Profiles of Cow’s Blood; a Review. J. Sci. Food Agric..

[47] Marino R., Braghieri A., Albenzio M., Caroprese M., Girolami A., Santillo A., Sevi A. (2009). Effect of Rearing System and of Dietary Protein Level on Leptin, Growth, and Carcass Composition in Young Podolian Bulls. J. Anim. Sci..

[48] Braghieri A., Pacelli C., De Rosa G., Girolami A., De Palo P., Napolitano F. (2011). Podolian Beef Production on Pasture and in Confinement. Animal.

[49] Ragni M., Colonna M.A., Lestingi A., Tarricone S., Giannico F., Marsico G., Facciolongo A.M. (2018). Effects of Protein Sources on Performance, Carcass Composition, Blood Parameters and Meat Quality in Charolais Heifers. S. Afr. J. Anim. Sci..

[50] Serrapica F., Masucci F., Romano R., Napolitano F., Sabia E., Aiello A., Di Francia A. (2020). Effects of Chickpea in Substitution of Soybean Meal on Milk Production, Blood Profile and Reproductive Response of Primiparous Buffaloes in Early Lactation. Animals.

[51] Coccodrilli G.D., Chandler P.T., Polan C.E. (1970). Effects of Dietary Protein on Blood Lipids of the Calf with Special Reference to Cholesterol. J. Dairy Sci..

[52] Park C.S., Fisher G.R., Haugse C.N. (1980). Effect of Dietary Protein and Sunflower Meal on Blood Serum Cholesterol of Dairy Heifers. J. Dairy Sci..

[53] Lee Y.H., Ahmadi F., Lee M., Oh Y.-K., Kwak W.S. (2020). Effect of Crude Protein Content and Undegraded Intake Protein Level on Productivity, Blood Metabolites, Carcass Characteristics, and Production Economics of Hanwoo Steers. Asian-Australas. J. Anim. Sci..

[54] Park C.S. (1985). Influence of Dietary Protein on Blood Cholesterol and Related Metabolites of Growing Calves. J. Anim. Sci..

[55] Silva L.F.C., Valadares Filho S.D.C., Chizzotti M.L., Rotta P.P., Prados L.F., Valadares R.F.D., Zanetti D., Braga J.M.D.S. (2012). Creatinine Excretion and Relationship with Body Weight of Nellore Cattle. Rev. Bras. Zootec..

[56] Freedland R.A., Szepesi B. (1971). Control of Enzyme Activity: Nutritional Factors1. Enzyme Synth. Degrad. Mamm. Syst..

[57] Bonanno A., Tornambè G., Grigoli A.D., Genna V., Bellina V., Miceli G.D., Giambalvo D. (2012). Effect of Legume Grains as a Source of Dietary Protein on the Quality of Organic Lamb Meat. J. Sci. Food Agric..

[58] Sommerfeldt J.L., Lyon K.A. (1988). Ration Digestibilities and Ruminal Characteristics in Steers Fed Chickpeas1. J. Dairy Sci..

[59] Gilbery T.C., Lardy G.P., Soto-Navarro S.A., Bauer M.L., Anderson V.L. (2007). Effect of Field Peas, Chickpeas, and Lentils on Rumen Fermentation, Digestion, Microbial Protein Synthesis, and Feedlot Performance in Receiving Diets for Beef Cattle. J. Anim. Sci..

[60] Antongiovanni M., Buccioni A., Petacchi F., Secchiari P., Mele M., Serra A. (2003). Upgrading the Lipid Fraction of Foods of Animal Origin by Dietary Means: Rumen Activity and Presence of Trans Fatty Acids and CLA in Milk and Meat. Ital. J. Anim. Sci..

[61] D’Alessandro A., Rinalducci S., Marrocco C., Zolla V., Napolitano F., Zolla L. (2012). Love Me Tender: An Omics Window on the Bovine Meat Tenderness Network. J. Proteom..

[62] Conte G., Serra A., Casarosa L., Ciucci F., Cappucci A., Bulleri E., Corrales-Retana L., Buccioni A., Mele M. (2019). Effect of Linseed Supplementation on Total Longissimus Muscle Lipid Composition and Shelf-Life of Beef From Young Maremmana Bulls. Front. Vet. Sci..

[63] Tocci R., Sargentini C., Tocci R., Sargentini C. (2020). Meat Quality of Maremmana Young Bulls. Acta Sci. Anim. Sci..

[64] Daghio M., Ciucci F., Buccioni A., Cappucci A., Casarosa L., Serra A., Conte G., Viti C., McAmmond B.M., Van Hamme J.D. (2021). Correlation of Breed, Growth Performance, and Rumen Microbiota in Two Rustic Cattle Breeds Reared under Different Conditions. Front. Microbiol..

[65] Maltin C., Balcerzak D., Tilley R., Delday M. (2003). Determinants of Meat Quality: Tenderness. Proc. Nutr. Soc..

[66] Christodoulou V., Ambrosiadis J., Sossidou E., Bampidis V., Arkoudilos J., Hucko B., Iliadis C. (2006). Effect of Replacing Soybean Meal by Extruded Chickpeas in the Diets of Growing–Finishing Pigs on Meat Quality. Meat Sci..

[67] Priolo A., Micol D., Agabriel J. (2001). Effects of Grass Feeding Systems on Ruminant Meat Colour and Flavour. A Review. Anim. Res..

[68] Kamilaris C., Dewhurst R.J., Vosough Ahmadi B., Crosson P., Alexander P. (2020). A Bio-Economic Model for Cost Analysis of Alternative Management Strategies in Beef Finishing Systems. Agric. Syst..

[69] Biagini L., Antonioli F., Severini S. (2020). The Role of the Common Agricultural Policy in Enhancing Farm Income: A Dynamic Panel Analysis Accounting for Farm Size in Italy. J. Agric. Econ..

[70] European Commission (EC) (2019). EU Agricultural Outlook for Markets and Income 2019–2030.

